# Supplementary material for: Contributions of Zea mays subspecies mexicana haplotypes to modern maize
Source: Nat Commun. 2017 Nov 30;8:1874. doi: 10.1038/s41467-017-02063-5 (PMC5707364; doi:10.1038/s41467-017-02063-5)
Supplement: Supplementary file 1 — Supplementary Information [file 41467_2017_2063_MOESM1_ESM.pdf]

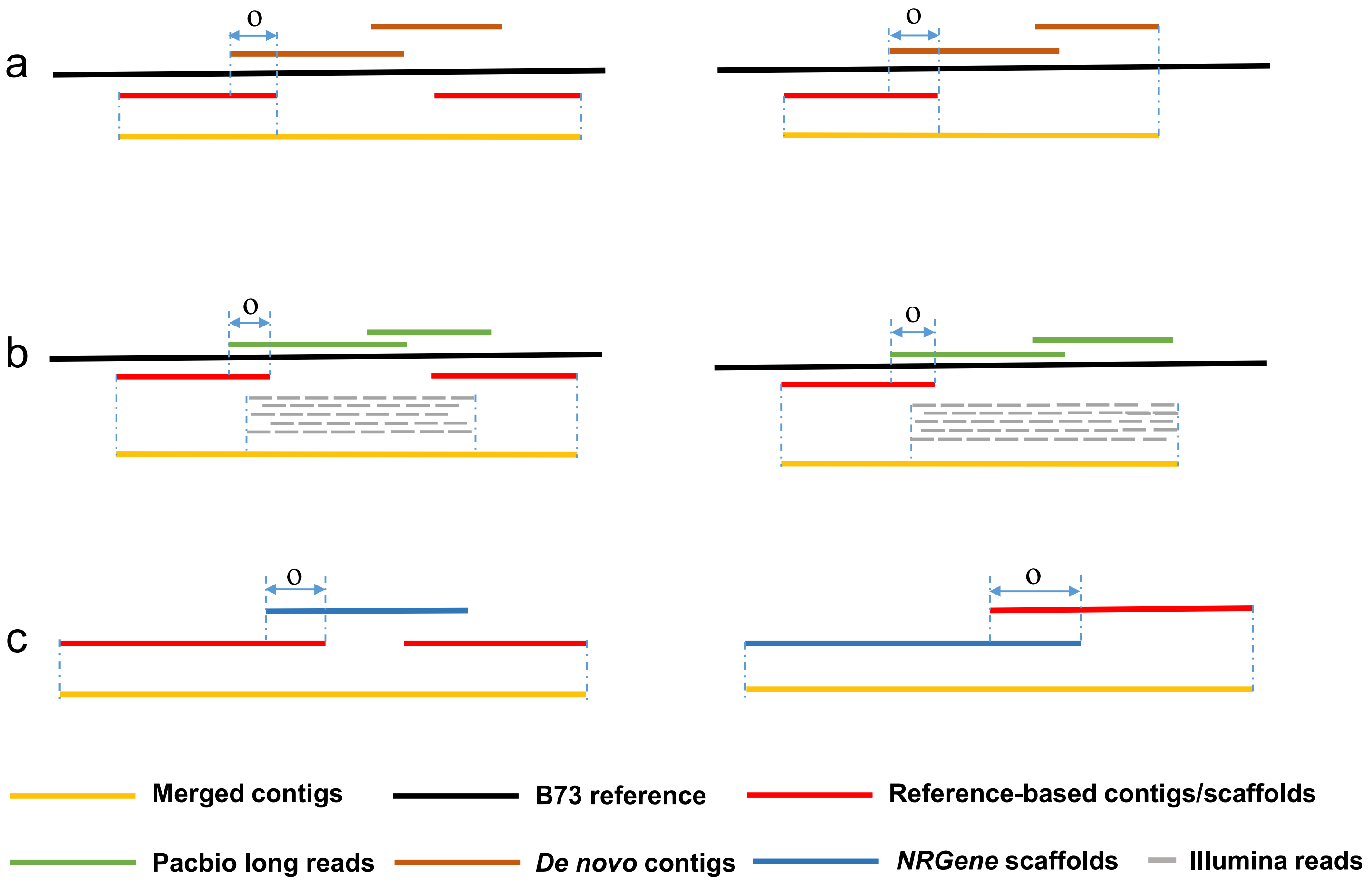

Supplementary Figure 1

Graphic representation of merging contigs and scaffolds. (a) Illustration of how to merge contigs from *de novo* assembly and reference-guided assembly. Black line indicates B73 reference, brown and red lines indicate the assigned contigs from *de novo* assembly and reference-guided assembly respectively, orange line indicates final merged contigs. Two reference-guided assembled contigs can be merged (left) or extended (right) if the overlap (o) between de novo assembled contigs and reference guided assembled contigs larger than 200 bp with 100%identity. **(b)** Green and gray lines indicate the assigned PacBio long reads and Illumina reads, respectively. If the overlap (o) between PacBio long reads and reference-guided assembled contigs larger than 200 bp with 100%identity, the Illumina reads in the gap region can be assembled with AMOS, and reference-guided contigs can be merged (left) or extended (right). **(c)** Graphic illustration of merging NRGene (red) and advanced (red) scaffolds. The matched sequence (o) should be ≥1 kb with identity ≥ 90%.

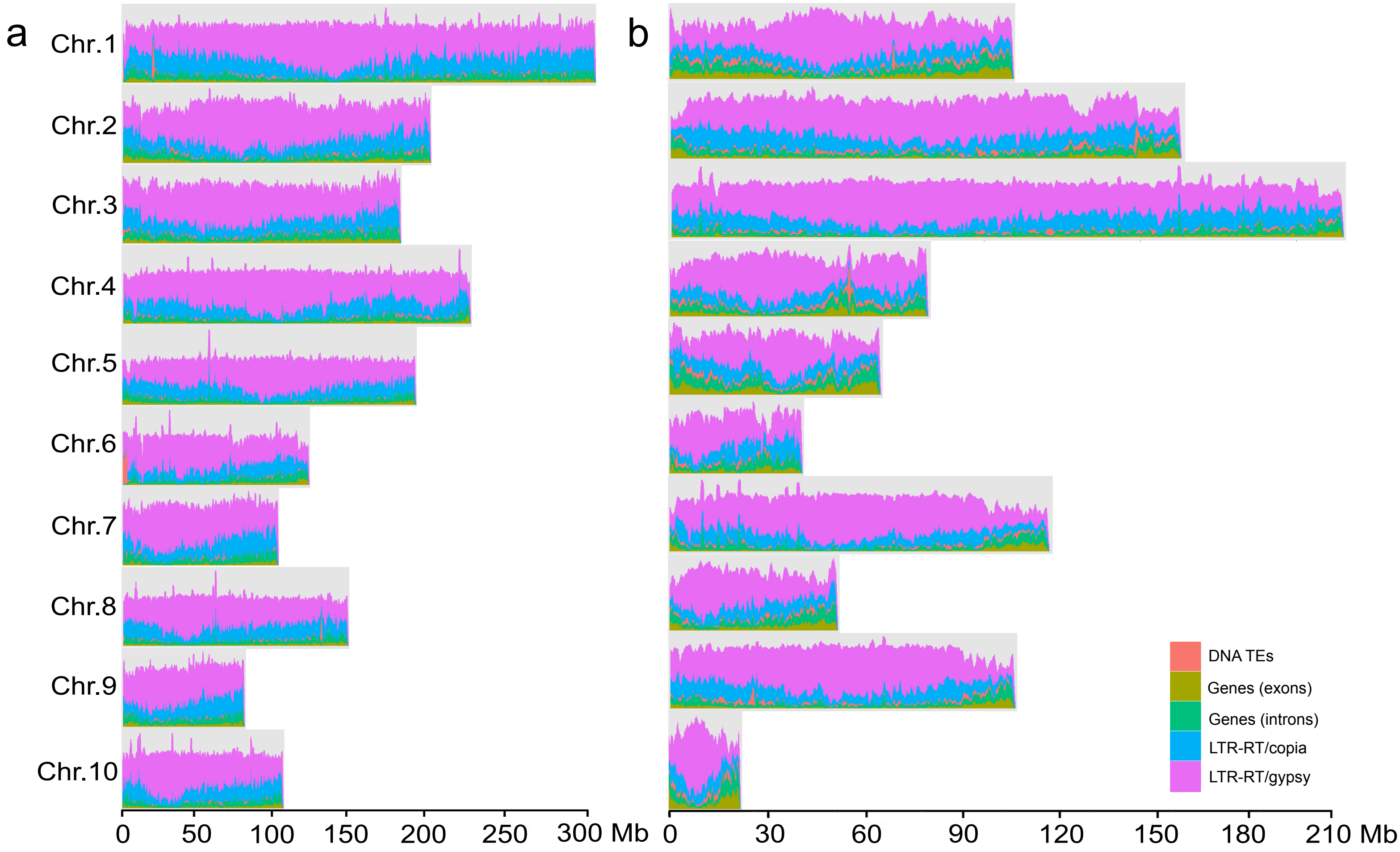

Supplementary Figure 2

Chromosomal distribution of the main (a) Mo17 and (b) *mexicana* genome features. Area charts quantify retrotransposons (*Copia* and *Gypsy*), genes (exons and introns), and DNA transposons. The *x* axis denotes the physical position along chromosomes in units of million bases (Mb).

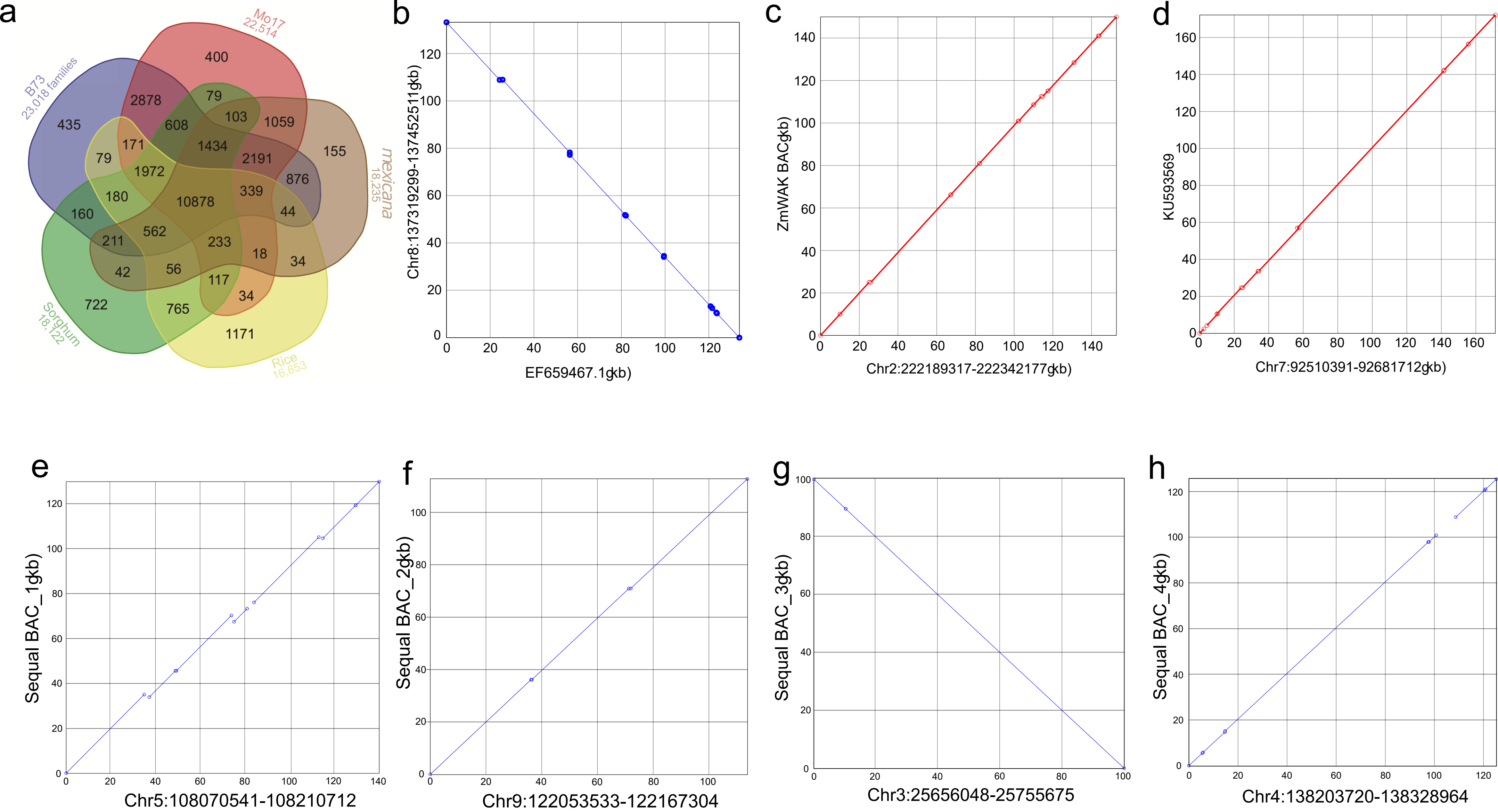

Supplementary Figure 3

Comparison of gene families among five plant species, and vlidation of the genome assembly for Mo17 and *mexicana*. (a) Unique and shared gene familes between B73, Mo17,*mexicana*,sorghum and rice are depicted in the 5-way Venn diagram. (b-h) Sequence comparison analysis between BAC sequences and Mo17 assembly. The *ZmWAK* BAC was available from Prof.Mingliang Xu's lab. And the other Mo17 BAC and scaffold sequences were downloaded from GenBank or assembled using Sequal sequences.

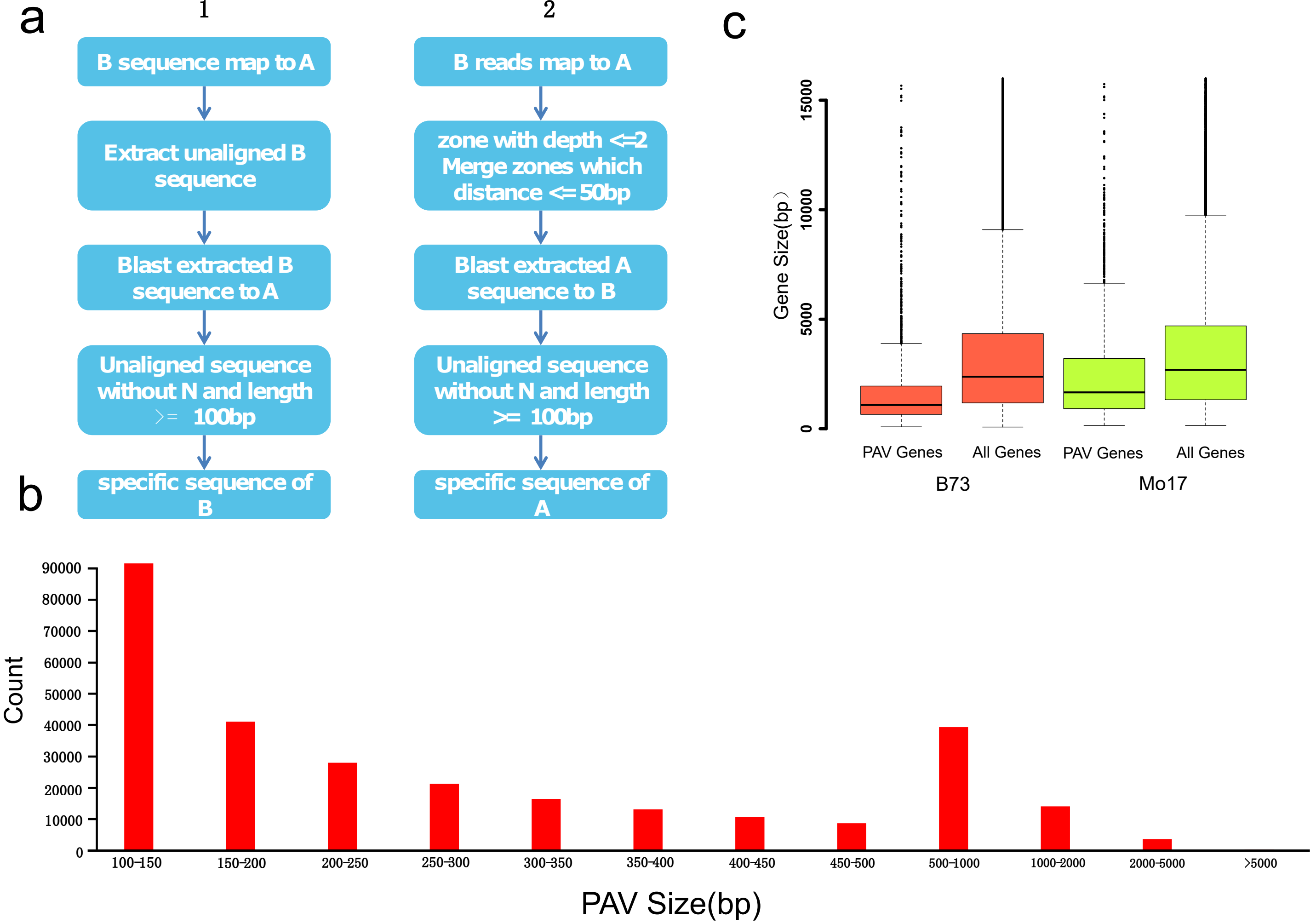

Supplementary Figure 4

The characterizations of PAVs from genome comparison. (a) PAV identification flowchart. Mo17 PAVs relative to B73: flowchart 1; B73 PAVs reltive to Mo17: flowchart 2; (b) Size distribution of identified PAVs compared wiht B73 genome. (c) Comparison of gene size for B73 /Mo17 genes and PAV genes.

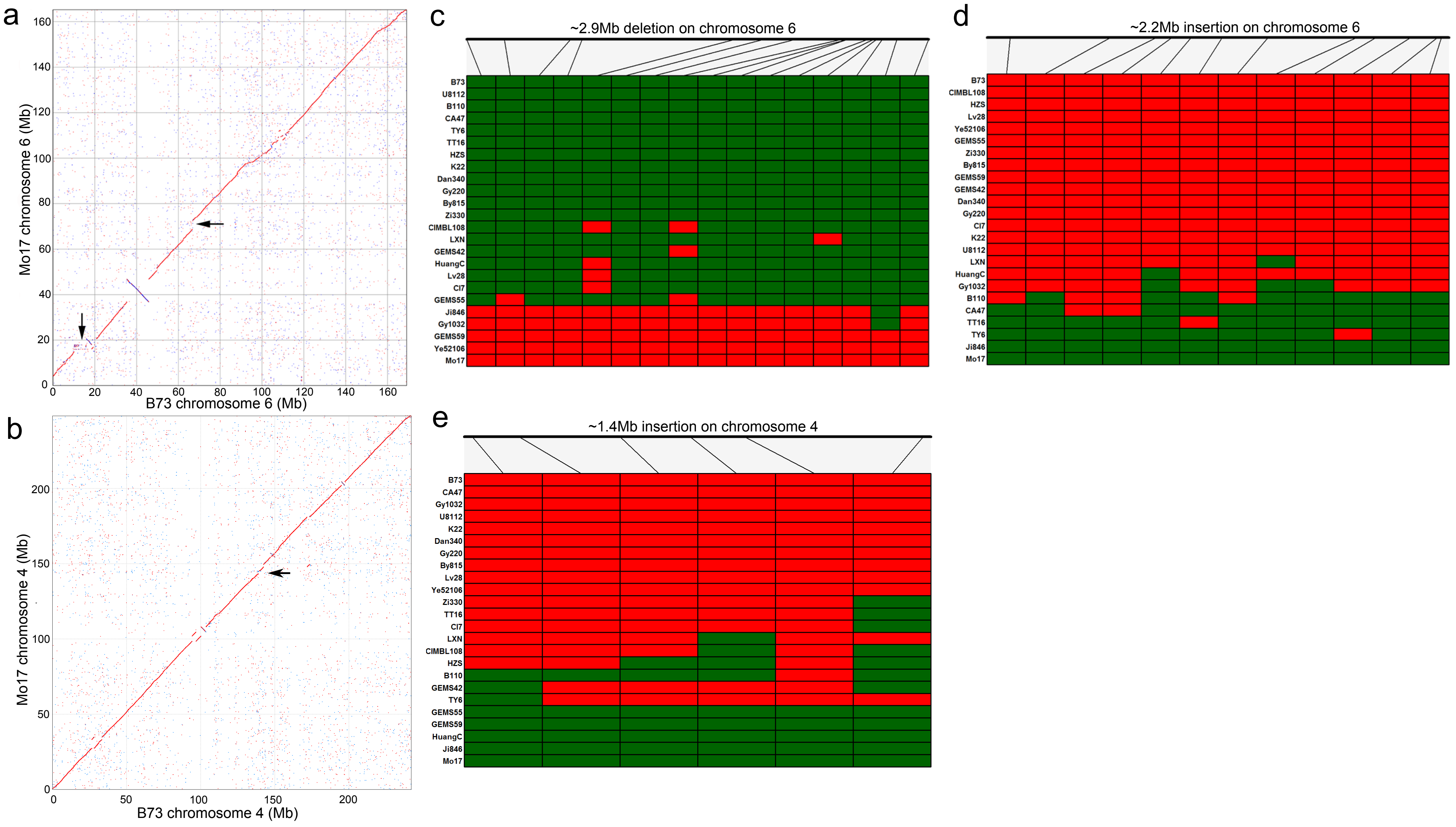

Supplementary Figure 5

Three striking mega-base-sized PAVs identified on chromosome 4 and chromosome 6 of Mo17 genome. (a, b) The dot-plot of genomic co-linearity between B73 and Mo17 on chromosomes 4 and 6. **(c-e)** The validation results from PCR amplifications. Green indicated successful amplification for a particular inbred by primer combination while red indicates no amplification.

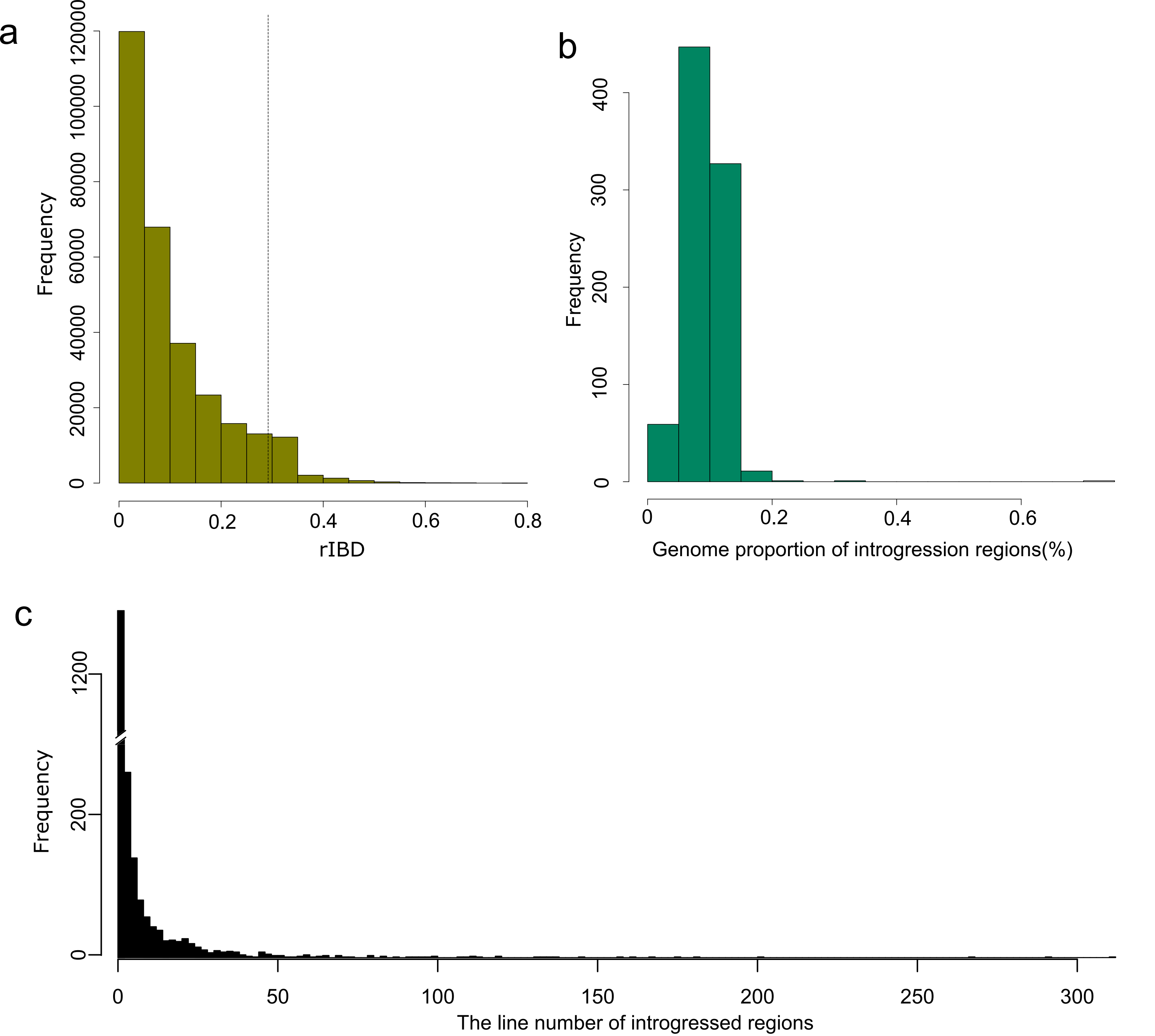

Supplementary Figure 6. (a) The selected introgression regions based on the statistic rIBD. the dash line indicated the threshold, the regions on the right were the final candidate introgression regions. **(b)** the distribution of introgression regions of each individual. **(c)** the distribution of the line number of introgressed regions.

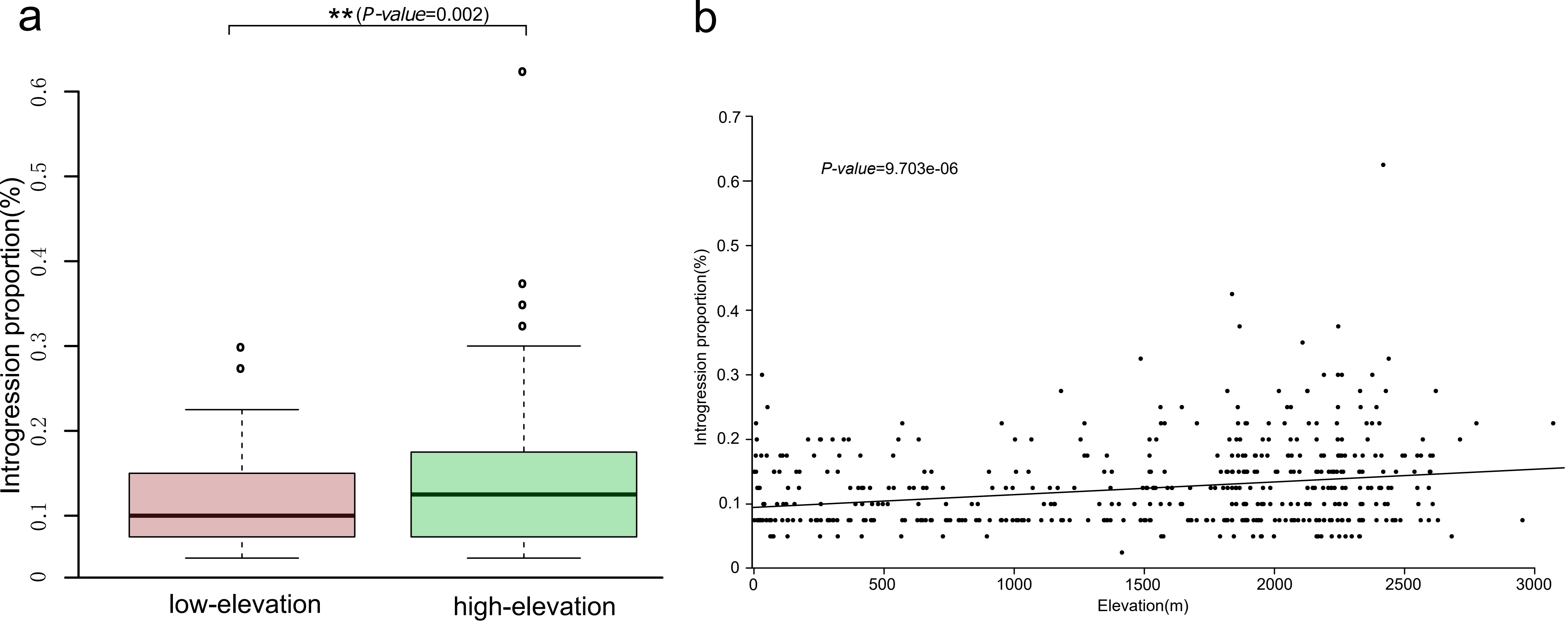

Supplementary Figure 7

The relationship of introgression proportion and elevation. (a) the introgression proportion had significantly difference between highlnd Mexico maize and lowland maize(low-elevation: <1,200m above sea level; high-elevation:>1,900m above sea level). **(b)** the positive correlation between elevation and introgression proportion.

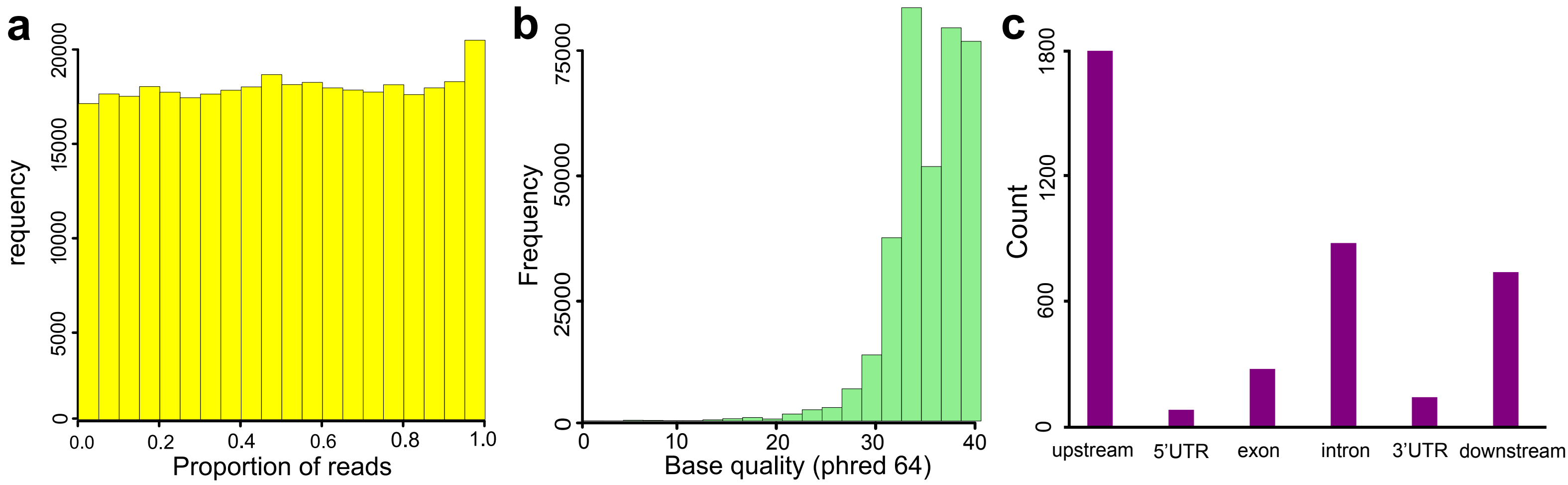

Supplementary Figure 8

Characterization of the supporting reads of mutations and the distribution of mutations across gene region. **(a)** Proportion of reads where the mutation genotype is found in the supporting reads. **(b)** The distribution of sequencing quality of the sites where the mutation genotype is found in the supporting reads. **(c)** The distribution of mutations across gene regions.

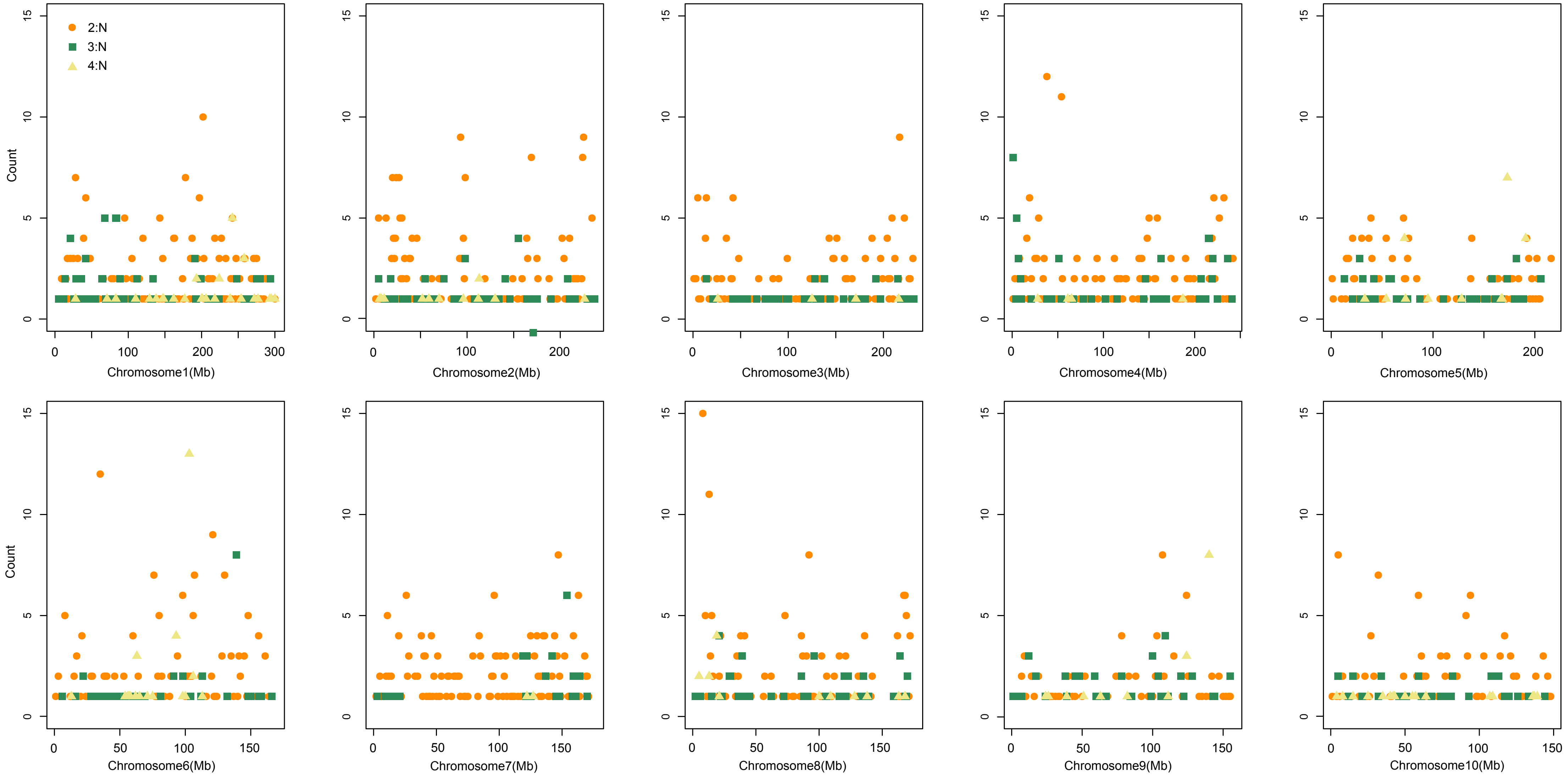

Supplementary Figure 9

The distribution of mutations detected in two or more individuals.

Supplementary Tables

Supplementary Table 1. The statistics of paired-end and mate-pair libraries

Lines	175bp (GB)	300bp (GB)	500bp (GB)	3K (GB)	9K (GB)	12K (GB)	Total coverage
TM3	41.01	38.38	32.48	6.38	5.81	11.22	67.63
TM24	30.9	34.66	30.65	5.4	6.07	5.23	56.46
TM52	38.35	39.77	32.27	6.21	9.26	7.55	66.71
TM89	38.05	0.26	58.53	10.69	6.58	4.67	59.39
TM104	37.44	37.71	32.45	9.61	12.26	10.61	70.05
TM117	40.75	33.45	31.15	8.54	5.22	4.82	61.96
TM148	30.64	30.18	35.51	8.21	6.34	6.7	58.79
TM183	39.75	34.65	32.48	7.7	8.56	7.95	65.55
TM186	27.63	0.76	51.31	8.46	6.69	8.63	51.74
TM192	36.78	35.56	31.09	8.23	6.7	7.59	62.98

Supplementary Table 2. PacBio libraries data statistics

Individuals	Average length(bp)	Max length(bp)	Reads number	Data size(GB)
TM24	7002	44,126	486,946	3.41
TM104	6059	33,885	498,817	3.02
Mo17	4764	30,661	600,564	2.86

Supplementary Table 3. NRGene libraries data statistics

Libraries(GB)	400-480bp	700-800bp	2-4kb	5-7kb	8-10kb
TM104	125.64	50.10	78.66	79.92	111.51

Supplementary Table 4. The classification and content of the repeat sequences

Classification			Mo17 (%)	*mexicana* (%)
**Transposable elements**	**Class I**	SINEs	0.07	0.10
		LINEs	0.81	0.85
		LTR elements	72.32	64.31
	**Class II**	DNA elements	4.84	5.38
**Unclassified**			0.77	1.25
**Other repeats**		Small RNA	0.06	0.13
		Satellites	0.46	0.35
		Simple repeats	0.39	0.50
		Low complexity	0.05	0.06
**Total**			79.67	72.79

Supplementary Table 5. Statistics of predicted gene models in Mo17 and *mexicana* genome

Genomes	I	Number	Total size (bp)	Mean length (bp)	GC content (%)
Mo17	Genes	40,003	195,287,155	4881.81	45.90
	Transcripts	97,069	215,461,277	2219.67	49.48
	Exons	681,597	215,461,277	316.11	49.48
	CDSs	97,069	127,175,785	1310.15	51.41
	UTRs	215,488	88,285,492	409.70	46.70
	Introns	584,528	402,820,101	689.14	42.85

Genomes	II	*ab initio* support[a]	Protein support[b]	EST support[c]	RNA-seq support[d]
Mo17	Genes level	32,776 (81.9%)	31,791 (79.5%)	26,491 (66.2%)	30,709 (76.8%)
	Transcript level	80,205 (82.6%)	83,878 (86.4%)	80,379 (82.8%)	77,147 (79.5%)
		**Protein/EST**	**EST/RNA-seq**	**Protein/EST/RNA-seq**	
	Genes level	23,946 (59.9%)	24,187 (60.5%)	22,018 (55.0%)	
	Transcript level	75,432 (77.7%)	68,130 (70.2%)	64,041 (66.0%)	

Genomes	I	Number	Total size (bp)	Mean length(bp)	GC content (%)
mexicana	Genes	31,387	128,559,131	4095.94	44.78
	Transcripts	71,535	147,752,925	2065.46	49.01
	Exons	480,989	147,752,925	307.19	49.01
	CDSs	71,535	89,829,859	1255.75	50.83
	UTRs	155,399	57,923,066	372.74	46.18
	Introns	409,454	233,610,750	570.54	40.84

**II**	***ab initio* support[a]**	**Protein support[b]**	**EST support[c]**	**RNA-seq support[d]**
Genes level	25,879 (82.5%)	22,830 (72.7%)	22,590 (72.0%)	26,715 (85.1%)
Transcript level	59,451 (83.1%)	58,526 (81.8%)	61,216 (85.6%)	61,680 (86.2%)
	**Protein/EST**	**EST/RNA-seq**	**Protein/EST/RNA-seq**	
Genes level	18,540 (59.1%)	21,168 (67.4%)	17,452 (55.6%)	
Transcript level	53,947 (75.4%)	54,869 (76.7%)	48,371 (67.6%)	

[a]*ab initio* support criterion: supported by at least on predictors (from FgenesH, Augustus & SNAP prediction results): ⩾ 50% number of exons

[b]Protein support criterion: coverage≥80%, identity ≥ 30% (from exonerate results)

[c]EST filtering criterion: identity≥ 75%, coverage ≥ 80% (from exonerate results)

[d]RNA-seq support criterion (calculate by RSEM): FPKM≥ 0.5

Supplementary Table 6. The statistics of the evaluation of Mo17 and *mexicana* assembled genomes

	BUSCO	CEGMA		CoreGF
	Complete (%)	Complete (%)	Partial (%)	Weighted score (%)
Mo17	93	88.7	96.0	94.0
mexicana	86	83.1	90.7	87.6
B73(V2)	93	87.9	95.6	96.1

Supplementary Table 7. The PAVs obtained by comparing the three genomes with each other

PAVs	B73&Mo17
Total Length	88,736,738
Length⩾500bp	50,929,442
Length⩾1000bp	29,239,087
Max length	105,644
Number of PAVs	220,860
PAV Genes number	1,293
Full length LTR	44
Number of TE-related PAVs	79,867 (36.16%)

Supplementary Table 8. The details of primers for the validation of the three megabase-sized structural variations

Structure Variations	Primers	Forward (5'-3')	Reverse (5'-3')
I	**Primer1_ 40000**	CGACGAGTTTGAGGATTAGG	CGAACAACCGACTCAGAAC
	**Primer2_ 178876**	CCCTGCTCATCATCTTGCT	AGGCTTTCAGGGATTGGA
	**IDP5870_ 263436**	TTCGACCTGACTCATCAGACC	GAAGCTGGGTCGTATTCTGC
	**IDP7015_ 665840**	CTTACCACAAGGCCCAAACC	AGCATCTTTGCTTGCTTTGC
	**IDP1993_ 740181**	GAAGACACCAACAGCATTCG	TGTGAAACAATGGCAGAAGC
	**IDP5958_ 1680079**	GGTTGATGTTCTACGGTGGG	GTCTTCCAACCGATCTTCTGC
	**IDP4398_ 1855132**	TCGGCAACTTCGTTTAGAGC	AACAATGCTTCTCCATTGCC
	**IDP5013_ 2035832**	ATCTGTGCGTCCTTTATCGG	TCGAGTGAAACAGCTCTTCG
	**IDP5899_ 2315981**	TTCGACCTGACTCATCAGACC	GAAGCTGGGTCGTATTCTGC
	**IDP4178_ 2353693**	CTGACAGCGTGATGTTACGG	TGTTGGCTTCCTTCTCAACG
	**IDP2010_ 2353693**	ATGATCAGCCTAACGCTTGC	TTGTGATGCATCTCGACTGG
	**IDP7930_ 2482061**	AGTCCTCATTCATGCCAAGG	GACGAGTGCTCTCAGTCACG
	**IDP7839_ 2522876**	TGACCATAAGGGACCAGACC	CCATGACTCTTTCTGCCTCC
	**IDP6876_ 2536813**	TGAATTGCAGCAAGATCAGC	TCAAGCTCGACAGATGATGG
	**IDP8422_ 2577483**	GTCTGGACCAAACCTCTTGC	TACGACACATCTGTGGGAGG
	**Primer3_ 2696086**	TGCTGTTGCCTCTGACGA	CAGGGCTCATTCCCAAAT
	**Primer4_ 2863654**	GGGTTGACGGCAGGTATT	TGGAAGAACGAGCCGAAG
II	**Primer1_114516**	AGACTCGACGATGAACGC	AAGCCTCGCCTCCTCCAT
	**Primer2_590457**	TCCCAGCCATCCACAGAA	AGTGTATTTAGGTGCGGGAG
	**Primer3_825858**	TTTCTGCGGCTCCCTTTA	GTGGCAATAGTAGAAGACAACG
	**Primer4_897469**	TCGGTAGCATGTGCATTG	AACGCAATACTAACTAAGGTCA
	**Primer5_990293**	CACCAACACGACTAACCCTT	CGACCCTATCTGTCTACGAACT
	**Primer6_1143394**	TCTCCGTGAATGGTGCTG	TCATAATCGAACGCTCCC

Structure Variations	Primers	Forward (5'-3')	Reverse (5'-3')
	**Primer7_1367932**	TACGCCGTCCTCATCCTT	CTACGATGGCAGGAACAG
	**Primer8_1792849**	AACAAATGGATGGGCACG	GGTTGAAATCATCCCAAAGG
	**Primer9_1889257**	GGGTGAAGGCATAATCCG	GCTTCCAGCTTTCCCTAG
	**Primer10_2057936**	GGGGCACCTCGTCATCTT	CGCAAACTCTAGGCAAGG
	**Primer11_2192451**	TCGTCCATGCAGACAACC	CTGCCACTGTCAATTCAAAC
	**Primer12_2204293**	ATTAAATCCGACTTGAAACG	CCTGGCTTCCTGCTAACC
Ⅲ	**Primer1_14244**	TCGATTAGACGGATGCTACG	GAACTCCACCCTGGCTCTT
	**Primer2_145611**	GCTTGCTACCGCCGAGAA	ACTCGTGCCGTCATGGTC
	**Primer3_390190**	GTATTCCGGCCCACAACT	GGACCTACTGACCGCAAA
	**Primer4_588710**	TACCGCATGGATTGGCTAG	CAGCGATCTGAACTGTGGG
	**Primer5_718220**	ATTGATGGAGCGGAGGGA	TTGAGATGGGTGGTGGAG
	**Primer6_1189760**	CTTGGGCTTGTGCTGGAA	TCGAAATCCCTTGGAAGC

Supplementary Table 9. The annotated genes of the two Mb-size insertions on Mo17 genome

Gene	chromosome	Position	Expressed	Annotation
ZEAMMMO17_027430	6	69926647	Kernel	Putative nuclease HARBI1
ZEAMMMO17_027431	6	69978165	No	Guanylate kinase
ZEAMMMO17_027438	6	71098558	No	5'-phosphate decarboxylase
ZEAMMMO17_027429	6	69741477	Kernel	Phosphopantetheine Adenylyltransferase
ZEAMMMO17_027434	6	70569083	No	N-alpha-acetyltransferase 11
ZEAMMMO17_027432	6	69990736	No	Alanine--tRNA ligase
ZEAMMMO17_027433	6	70375205	No	Dirigent protein 11
ZEAMMMO17_027437	6	71022774	No	Dirigent protein 23
ZEAMMMO17_027435	6	70973242	No	Uncharacterized protein
ZEAMMMO17_027436	6	70977096	No	Hypothetical protein
ZEAMMMO17_020101	4	144938134	All	G protein beta WD-40 repeat
ZEAMMMO17_020100	4	144932122	All	Thiosulfate/3-mercaptopyruvate Sulfurtransferase 1
ZEAMMMO17_020099	4	144779228	No	Magnesium/proton exchanger 2
ZEAMMMO17_020098	4	144634759	No	Homeobox protein prospero homolog 1
ZEAMMMO17_020097	4	144630438	No	Light regulated Lir1
ZEAMMMO17_020096	4	144502878	Young leaf, mature leaf	Serine/Threonine protein kinases
ZEAMMMO17_020095	4	144387524	No	Uncharacterized protein
ZEAMMMO17_020094	4	144092539	No	Uncharacterized protein

Supplementary Table 10. The point mutation rate of different chromosomes

Chromosomes	Number of mutations	Chromosome length	Mutation rate
Chr1	1,085	301,354,135	3.60×10^{-8}
Chr2	1,122	241,473,504	4.65×10^{-8}
Chr3	1,037	237,068,873	4.37×10^{-8}
Chr4	841	232,140,174	3.62×10^{-8}
Chr5	718	217,872,852	3.30×10^{-8}
Chr6	586	176,764,762	3.32×10^{-8}
Chr7	760	175,793,759	4.32×10^{-8}
Chr8	574	169,174,353	3.39×10^{-8}
Chr9	568	156,750,706	3.62×10^{-8}
Chr10	669	150,189,435	4.45×10^{-8}
Total	7,960	2,058,582,553	3.87×10^{-8}

Supplementary Table 11. Summary of point mutations

	Total	Gene	Intergenic	Centromeres
Total number	7,960	3,932	4,028	1,310
Region size(Mb)	2,058,582,553	161,205,073	1,897,377,480	439,656,844
Mutation rate	3.87×10^{-8}	8.84×10^{-8}	2.50×10^{-8}	2.98×10^{-8}
AT>GC	1,775	911	864	264
GC>AT	3,800	1,813	1,987	627
AT>CG	430	231	199	58
AT>TA	526	256	270	89
GC>TA	976	452	524	201
GC>CG	453	269	184	71
Transitions/transversions	2.34	2.25	2.42	2.13
GC>AT/AT>GC	2.14	1.99	2.30	2.38

Supplementary Table 12. The five miss-scaffolding scaffolds by NRGene

Variation Type	Chromosome[a]	Start Position[b]	End Position[c]	Variation Length
Insertion	1	194665481	194666505	7516260
Insertion	5	14231725	14359255	1756983
Deletion	6	62352849	66854821	4501972
Deletion	6	10087054	16008215	5921161
Insertion	10	94953411	94952513	1846672

[a-c] The relative B73 physical position of the mis-assembled scaffolds.

Supplementary Table 13. The SNPs heterozygosity rate in 10 lines and the cross-validation between SNPs from our SNP calling pipeline and Axiom Maize Genotyping Array

Lines	Consistent number	Co-location	Ratio (%)	Het Number	Total Number	Het Rate (%)
TM3	164693	164870	99.89	53872	3232340	1.67
TM24	131757	132651	99.33	146282	3101312	4.72
TM52	134906	135818	99.33	167213	3359973	4.98
TM89	130546	130929	99.71	40239	3074563	1.31
TM104	131750	132039	99.78	31942	3088205	1.03
TM117	140017	140146	99.91	17638	3146453	0.56
TM148	129121	129360	99.81	23114	2969514	0.78
TM183	136849	137020	99.88	25037	3169494	0.79
TM186	99859	100046	99.81	8454	2075090	0.41
TM192	48611	49901	97.41	135821	2383253	5.70
Average			99.49			2.20
Mo17	169807	170472	99.61	40575	3261877	1.24
mexicana	157488	158486	99.37	118251	3911448	3.02

Supplementary Note

Supplementary Note 1

Contigs assembly using Illumina reads and PacBio long reads

The filtered paired-end reads were corrected using SOAPec[1] (V2.01) with default parameters. The paired-end and mate-pair libraries were assigned to each bin showed in Fig. 1b based on the B73 reference genome by using bwa[2] (V0.7.4) and NovoAlign (V3.02.05) (http://www.novocraft.com/products/novoalign/) with default parameters. The filtered long PacBio reads were aligned to the maize B73 genome[3] using BLASR[4].

We employed three strategies to assembly contigs, including *de novo* assembly of ten individuals (strategy 1), reference-based assembly based on B73 genome (strategy 2) and *de novo* assembly of unmapped reads (strategy 3). Strategy 1: For each of the ten individuals, whole genome *de novo* assembly was performed using SOAPdenovo2[1] (V2.04). The K-mer size was set to {49, 59, 63, 69, 79, 89} (-K 49, 59, 63, 69, 79, 89), and read repeat resolution (-R) was enabled. The assembled contig N50 for the ten individuals was in the range of 522~2,549 bp. Strategy 2: Reference-based assembly in each of 211 bins (Mo17) and 176 bins (*mexicana*) was performed using MaSuRCA (V.2.1.0)[5] with default parameters, and the assembled contig N50 for Mo17 and *mexicana* was 2,478 bp and 3,175 bp respectively. Strategy 3: The unmapped reads can be divided into Mo17 and *mexicana* reads according to the bin combination, and *de novo* assembled separately into Mo17 and *mexicana* contigs with N50 1,298 bp and 1,193 bp.

Then we merged the contigs of the above three strategies, some *de novo* assembled contigs can connect two bin-based contigs, or extent bin-based contig (**Supplementary Fig. 2a)**. The length of overlap region between the merged or extended contigs must be larger than 200 bp with 100% identity. After merging the above contigs, Pacbio long reads can further connect or extend the contigs. Considering that the high error rate of Pacbio sequence, we didn’t used the Pacbio sequences to extend the contigs directly. Illumina read in extended or connected regions were used to assembly and extend contigs with AMOS[6] (**Supplementary Fig. 2b)**. After this step, contig N50 for Mo17 and *mexicana* was extended to 9,678 bp and 5,674bp, respectively.

The longer (5 kb, 9 kb, 12 kb) mate-pair libraries were used for scaffolding with SSPACE[7] on the output of contigs with default parameters and no contig extension (-x 1). After scaffolding, SOAP GapCloser[1] (V1.12) (-p 32, -l 96), GapFiller[8] and Pbjelly[9] (-x "--minGap=1") were used for bridging scaffold gaps with paired-end reads and PacBio long reads. In this step, the contig N50 for Mo17 and *mexicana* was extended to 24,312 bp and 11,657bp, and scaffold N50 for the two genomes was 138,269 bp and

28,634bp, respectively.

NRGene assembly for TM104

PCR duplicates, Illumina adaptor AGATCGGAAGAGC and Nextera linkers (for mate-pair libraries) were removed. For the 2x250, 450 bp paired-end (PE) libraries overlapping reads were merged with minimal required overlap (10 bp) to create the stitched reads. Following pre-processing, all reads containing putative sequencing errors (containing a sub-sequence that does not reappear several times in other reads) were filtered. Genome was assembled using DenovoMAGIC 2^{TM} (http://nrgene.com/products-technology/denovomagic/). The first step of the assembly consisted of building a De Bruijn graph of contigs from the overlapping PE reads, and then the PE reads were used to find reliable paths between contigs in the graph for repeat resolution and contig extension. Contigs were linked to scaffolds with PE and mate-pair information, gaps between contigs were estimated according to the distance of PE and mate-pair links. The final gap-filling step used PE and mate-pair links and De Bruijn graph information to detect a unique path connecting the gap edges.

Combination of NRGene scaffolds and assembled scaffolds

Finally, the NRGene scaffolds and the assembled scaffolds were merged to build the final scaffolds. NRGene scaffolds were used to connect or extend the assembled scaffolds (**Supplementary Fig. 2c**). The final contig N50 for Mo17 and *mexicana* was 60,508 bp and 26,638 bp, and scaffold N50 for Mo17 and *mexicana* was 2,995,073 bp and 107,689 bp, respectively.

Supplementary Note 2

In order to anchor the scaffolds, a high-density genetic linkage map was developed using the TM population with 191 recombination inbred lines derived from a cross Mo17-*mexicana* (accession: PI566673) and genotyped with 56k SNP array. The genetic map spanned 1,748 cM and contained 1,282 bins derived from 12,390 high-quality SNPs. Firstly, scaffolds were ranked using B73 reference position by aligning the probes of 600k SNP array to scaffolds, and 592,202 and 498,056 probes were matched to Mo17 and *mexicana* scaffolds, respectively. Secondly, the genetic linkage map was used to adjust structure variations and misassembly of Mo17 and *mexicana* genomes, 1,973 (96.6%) Mb of the Mo17 genome and 1,072 (88.8%) Mb of the *mexicana* genome was anchored in this step. For the remaining scaffolds, 36.4 Mb (1.8%) of the Mo17 genome and 85.5 Mb (7.1%) of the *mexicana* genome were anchored using genotype by sequencing (GBS) probes.

Supplementary Note 3

The pipeline for gene prediction included *de novo* prediction on the repeat-masked genome and evidence-based predictions using PASA[10]. Three *de novo* gene predictors: Augustus[11], FgenesH[12], and SNAP[13] were employed for gene prediction. Augustus[11] and FgenesH[12] were used with the parameters set for maize, and SNAP[13] was conducted with the parameters set for rice. Consequently, Augustus[11] and FgenesH[12] were assigned greater weight than SNAP[13] in the integration. 7,571,071 EST sequences from all Poaceae plants were downloaded from NCBI. The assembled EST sequences from PlantGDB[14] were collected, including 181,717 sequences from maize, 518,012 sequences from rice, and 581,531 sequences from other monocots. Protein sequences include 547,328 sequences of all species that were obtained from the SwissProt database[15]; 670,693 sequences of all Poaceae plants that were obtained from the UniProt database[16], and 1,231,797 sequences of monocots that were obtained from NCBI, and sequences of annotated proteins of rice, *Arabidopsis*, sorghum, and maize. We also sequenced 30 RNA-seq libraries from three tissues of the 10 TM individuals using Illumina RNA-seq technology. The above data were filtered using the following two steps. (1) The redundancy of EST and protein sequences and the sequences containing unknown nucleotides or amino acids were filtered; (2) CD-HIT and CD-HIT-EST[17] were employed with parameter –c 1 to filter the protein sequences and ESTs separately. RNA-seq reads were aligned to Mo17 and *mexicana* genomes, the aligned reads were used as input for Trinity[18] for *de novo* transcript assembly, and for Cufflinks[19] for reference-based transcript assembly. *De novo* assembled transcripts were also filtered using the above procedures. All EST, *de novo* assembled transcripts and protein sequences were mapped to Mo17 and *mexicana* genomes (identity>80%) using Exonerate[20] to predict gene structures. *De novo* assembled transcripts and EST sequences were used as input for PASA15. All the predicted gene structures were combined into consensus gene models using EVM[21]. The output of EVM[21] was refined again by PASA[10] assembly alignments.

We obtained 75,161 and 65,963 candidate gene models for Mo17 and *mexicana*, respectively, and then filtered the gene models according to the following criteria: (1) Gene models annotated only by *ab initio* with no homologous proteins in NCBI nr database (coverage>=85%, identity>=85%, E-value<=1e-5) were removed; (2) Gene models contain >10% missing amino acids were removed; (3) Gene models were aligned to pfamA[22] using hmmscan[23] to filter TE-related genes; (4) Gene models without homologous proteins (coverage>=50%, identity>=50%, E-value<=1e-5) and RNA-seq evidence were removed.

Supplementary Note 4

Plant material, DNA extraction for paired-end and meta-pair libraries

Seeds of the TM population were sown in 2013 in Sanya, Hainan Province, China. Young leaves were collected and frozen at -80 ℃ for DNA extraction. DNA for paired-end libraries was extracted by a modified CTAB procedure for each line according to Murry and Thompson (1980)[24]. For mate-pair libraries, high molecular weight DNA extraction and purification was performed using a DNeasy Plant Maxi Kit (Qiagen, Germany). 0.8g of young leaves was ground to a fine powder in liquid nitrogen using a mortar and pestle and then transferred to a 15 mL centrifuge tube. The supplied 5 mL Buffer AP1 and 10 μl RNase A were added to the tube and mixed vigorously until there were no visible tissue clumps. The tube was then incubated at 65 ℃ for 60 minutes and gently inverted every 10 minutes during incubation. Buffers P3, AW1, and AW2 were added, followed by centrifugation, according to the kit's protocol. DNAase-free water was used for elution. DNA concentration was measured using Nanodrop (Thermo Fischer, Schwerte, Germany) and Qubit 2.0 (Invitrogen, Karlsruhe Germany).

Construction of illumina paired-end and mate-pair libraries

For short insert libraries, genomic DNA was sheared to 175-500 bp fragments using the Bioruptor Sonication System (Diagenode, USA). DNA was resolved on a 2% agarose gel at 120 V for 80 minutes. Then the fragments of approximately 300 bp, 420 bp, and 620 bp were selected and extracted with a Gel Extraction Kit (Qiagen, Germany). The isolated DNA was amplified by PCR for 10 cycles with the supplied PCR primer cocktail (Illumina) and cleaned up using the AMPure XP Beads (Beckman Coulter, USA). Validation of the libraries was performed using an Experion automated electrophoresis system (Bio-Rad, California) by running 1 μl sample on a DNA chip (Experion DNA Analysis Kits). The libraries were stored at -20 ℃.

Mate-pair libraries (insertion DNA fragments ranging from 5 kb to 15 kb) were prepared from purified high molecular weight DNA. For the long insertion libraries, genomic DNA was sheared to 2-20 kb fragments using the supplied mate-pair tagment enzyme. Strand displacement and purification using AMPure XP Beads were performed according to Illumina's Nextera mate-pair sample preparation guide. To select the target size of DNA fragments, DNA was resolved on a 0.6% Megabase agarose gel (Invitrogen, Karlsruhe Germany) at 100 V for 80 minutes. The fragments of approximately 5 kb, 10 kb, and 15 kb were selected and isolated with a Zymoclean large fragment DNA recovery kit (Zymo Research Corporation, USA). DNA size selection was performed by circularization for 16 hours overnight at 30 ℃. After circularization, the remaining linear DNA was digested by exonuclease and the circularized DNA was sheared using the Bioruptor Sonication System (Diagenode, USA). The sheared DNA was bound to the supplied streptavidin beads. Next, DNA fragments containing the biotinylated junction adapters were purified by binding to

streptavidin magnetic beads, the unbiotinylated molecules were removed through washing. End repair of sheared fragments, addition an adenylate to 3' ends, and ligation of indexed paired-end adapters was performed as described for paired-end libraries. All samples were processed on beads. After ligation with the adapters, DNA was amplified by PCR for 15 cycles with the supplied PCR primer cocktail (Illumina) and cleaned up using the AMPure XP Beads (Beckman Coulter, USA). Validation and storage of the libraries were performed as for paired-end libraries.

Illumina and PacBio DNA sequencing

The concentration of final libraries was measured with qPCR (Bio-Rad, California). Cluster generation was performed on a cBot (program: PE_Amp_Lin_Block_Hyb_v8.0, Illumina) using a flow cell v3 and reagents from TruSeq PE Cluster Kits v3 (Illumina) according to the manufacturer's instructions. DNA was subjected to paired-end sequencing on a HiSeq2000 equipped with on-instrument HCS version 1.4.8 and real time analysis (RTA) version 1.12.4.2 (Illumina).

The 20 kb SMRT cell sequencing libraries (Pacific Biosciences) were constructed for Mo17, TM24, and TM104 individuals following the protocol described in Quail et al[25]. Each library was sequenced with 10 SMRT cells (Pacific Biosciences) using PacBio P5 binding kit and C3 sequencing kit. Libraries were loaded by magbeads mode and 1 ×180 minute movies were captured for each SMRT cell using the PacBio RS II (Pacific Biosciences) sequencing platform. Primary filtering was performed on RS Blade Center server and secondary analysis was performed using the SMRT analysis pipeline[26] version 1.4.

Reads quality control and library insertion size estimation

The paired-end libraries were trimmed using Trimmomatic[27] (v0.30) to remove Illumina adapters and low-quality bases. TruSeq3 paired-end adapter sequences supplied with Trimmomatic[27] were used to remove adapters. Low-quality bases (quality score below 3) were removed from both ends of the reads, then the sliding window trimmer was used to remove low-quality sequences on the 3' end, using an average quality score of 20 over 4 bases. Reads shorter than 90 bp were filtered. The parameters were as follows:

- ILLUMINACLIP: TruSeq3-PE.fa:2:30:10
- LEADING: 3
- TRAILING: 3
- SLIDINGWINDOW: 4:20
- MINLEN: 90

Duplicates of paired-end reads introduced by PCR amplification were removed using fastUniq[28].

For mate-pair libraries, adapters were first removed using cutadapt (V1.3)

(http://cutadapt.readthedocs.org/en/stable/), and then using Trimmomatic[27] (V0.30) to remove low-quality reads with the same parameters.

For PacBio long reads, TrimmingReads.pl was used to trim 20 bases from the beginning of the reads and reads shorter than 1 kb were filtered.

• perl TrimmingReads.pl -l 20 -n 1000

The library statistics are listed in **SupplementaryTable 1-3**. Reads of all libraries were aligned using bwa[2] (V0.7.4) against the maize B73 reference[3] V2.5b. The CollectInsertSizeMetrics function in the Picard package (http://picard.sourceforge.net) was used to estimate insertion size.

Supplementary References

1. Luo, R.B. *et al*. SOAPdenovo2: an empirically improved memory-efficient short-read de novo assembler. *Gigascience* **1**, 18 (2012).
2. Li, H. & Durbin, R. Fast and accurate short read alignment with Burrows-Wheeler transform. *Bioinformatics* **25**, 1754-1760 (2009).
3. Schnable, P.S. *et al.* The B73 maize genome: complexity, diversity, and dynamics. *Science* **326**, 1112-1115 (2009).
4. Chaisson, M.J. & Tesler, G. Mapping single molecule sequencing reads using basic local alignment with successive refinement (BLASR): application and theory. *BMC Bioinformatics* **13**, 238 (2012).
5. Zimin, A.V. *et al*. The MaSuRCA genome assembler. *Bioinformatics* **29,** 2669-2677 (2013).
6. Schatz, M.C. *et* a*l*. Hawkeye and AMOS: visualizing and assessing the quality of genome assemblies. *Brief Bioinform.* 14, 213-224 (2013).
7. Boetzer, M., Henkel, C.V., Jansen, H.J., Butler, D. & Pirovano, W. Scaffolding pre-assembled contigs using SSPACE. *Bioinformatics* **27**, 578-579 (2011).
8. Nadalin, F., Vezzi, F. & Policriti, A. GapFiller: a *de novo* assembly approach to fill the gap within paired reads. *BMC Bioinformatics* **13**, S8 (2012).
9. English, A.C. *et al*. Mind the gap: upgrading genomes with Pacific Biosciences RS long-read sequencing technology. *Plos One* **7**, e47768 (2012).
10. Haas, B.J. *et al*. Improving the *Arabidopsis* genome annotation using maximal transcript alignment assemblies. *Nucleic Acids Res.* **31**, 5654-5666 (2003).
11. Stanke, M. & Morgenstern, B. AUGUSTUS: a web server for gene prediction in eukaryotes that allows user-defined constraints. *Nucleic Acids Res.* 33, W465-W467 (2005).
12. Salamov, A.A. & Solovyev, V.V. *Ab initio* gene finding in *Drosophila* genomic DNA. *Genome Res.* 10, 516-522 (2000).
13. Johnson, A.D. *et al*. SNAP: a web-based tool for identification and annotation of proxy SNPs using HapMap. *Bioinformatics* 24, 2938-2939 (2008).

14. Duvick, J. *et al*. PlantGDB: a resource for comparative plant genomics. *Nucleic Acids Res.* 36, D959-D965 (2008).
15. Boutet, E. *et al*. UniProtKB/Swiss-Prot, the manually annotated section of the UniProt knowledgebase: how to use the entry view. *Methods Mol. Biol.* 1374, 23-54 (2016).
16. UniProt Consortium. UniProt: a hub for protein information. *Nucleic Acids Res.* **43,** D204-D212 (2015).
17. Fu, L., Niu, B., Zhu, Z., Wu, S. & Li, W. CD-HIT: accelerated for clustering the next-generation sequencing data. *Bioinformatics* 28, 3150-3152 (2012).
18. Grabherr, M.G. *et al*. Full-length transcriptome assembly from RNA-Seq data without a reference genome. *Nat. Biotechnol.* 29, 644-652 (2011).
19. Trapnell, C. *et al*. Transcript assembly and quantification by RNA-Seq reveals unannotated transcripts and isoform switching during cell differentiation. *Nat. Biotechnol.* 28, 511-515 (2010).
20. Slater, G.S. & Birney, E. Automated generation of heuristics for biological sequence comparison. *BMC Bioinformatics* 6, 31(2005).
21. Haas, B.J. *et al*. Automated eukaryotic gene structure annotation using EVidenceModeler and the program to assemble spliced alignments. *Genome Biol.* **9**, R7 (2008).
22. Finn, R.D. *et al*. Pfam: the protein families database. *Nucleic Acids Res.* 42, D222-D230 (2014).
23. Mistry, J., Finn, R.D., Eddy, S.R., Bateman, A. & Punta, M. Challenges in homology search: HMMER3 and convergent evolution of coiled-coil regions. *Nucleic Acids Res.* 41, e121 (2013).
24. Murry, M.G. & Thompson, W.F. Rapid isolation of high molecular weight plant DNA. *Nucleic Acids Res*. 8, 4321–4325 (1980).
25. Quail, M.A. *et al*. A tale of three next generation sequencing platforms: comparison of Ion Torrent, Pacific Biosciences and Illumina MiSeq sequencers. *BMC Genomics* 13, 341 (2012).
26. Chin, C.S.*et al*. Nonhybrid, finished microbial genome assemblies from long-read SMRT sequencing data. *Nat. Methods* 10, 563-569 (2013).
27. Bolger, A.M. Lohse, M. & Usadel, B. Trimmomatic: a flexible trimmer for Illumina sequence data. *Bioinformatics* 30, 2114-2120 (2014).
28. Xu, H. *et al*. FastUniq: a fast *de novo* duplicates removal tool for paired short reads. *Plos One* **7**, e52249 (2012).
