# Supplementary material for: Contributions of Zea mays subspecies mexicana haplotypes to modern maize
Source: Nat Commun. 2017 Nov 30;8:1874. doi: 10.1038/s41467-017-02063-5 (PMC5707364; doi:10.1038/s41467-017-02063-5)
Supplement: Supplementary file 3 — Description of Additional Supplementary Files [file 41467_2017_2063_MOESM3_ESM.pdf]

Description of Supplementary Files

File Name: Supplementary Data 1
Description: The position and origin information of *mexicana* bins used for the meta-assembling.

File Name: Supplementary Data 2
Description: The position and origin information of Mo17 bins used for the meta-assembling.

File Name: Supplementary Data 3
Description: The rate of putative introgression regions between maize and *mexicana* for the individuals of maize hapmap3.

File Name: Supplementary Data 4
Description: The overlap between putative introgression regions and detected QTLs in TM population.
